# Supplementary material for: Mediterranean Diet Adherence in Celiac Patients: A Nested Cross-Sectional Study
Source: Nutrients. 2025 Feb 25;17(5):788. doi: 10.3390/nu17050788 (PMC11901495; doi:10.3390/nu17050788)
Supplement: Supplementary file 1 [file nutrients-17-00788-s001.zip › nutrients-3494188-supplementary.pdf]

Table S1.

ID Number: ________

<u>MEDITERRANEAN DIET SCORE (MDS) QUESTIONNAIRE</u>

	Score items	Units	1 point	0.5 point	0 point
1.	Vegetables (without potatoes)	Servings per day	≥3	1-3	<1
2.	Legumes and nuts	Portions per week	≥2	1-2	<1
3.	Fruits	Servings per day	>2	1-2	<1
4.	Whole grain cereals	Servings per day	≥2	1-2	<1
5.	Lean meat	Servings per week	>4	2-4	<2
6.	Fish	Servings per week	>2	1-2	<1
7.	Fatty meat and processed meat	Servings per week	<1	1-2	>2
8.	High-fat, not fermented dairy products	Servings per day	<1	1-2	≥2
9.	Low-fat and fermented dairy products	Servings per day	≥2	1-2	<1
10.	Vegetable oils	Teaspoons per day	4-8	2-4	>8 or <2
11.	Olive and canola oil	Teaspoons per day	>3	1-3	<1
12.	Avocado	Units per week	>3	0,5-3	<0,5
13.	Sugar	Teaspoons per day	<4	4-8	>8
14.	Wine	Glasses per day	Women: ≤1<br>Men: ≤2<br>Moderate and usually with meals	Women: ≤1<br>Men: ≤2<br>Moderate and rarely with meals	Women: >1<br>Men: >2<br>or non-drinkers

Table S2. Adequate and inadequate Mediterranean diet adherence in controls, new CD patients and CD patients on a GFD

		**CD on GFD n=128**	**New CD n=24**	**Controls n=63**	**CD on GFD vs. new CD**	**CD on GFD vs. controls**	**New CD vs. controls**
						***p*-value**	
**Vegetables** n (%)	0	111 (87%)	20 (83%)	50 (80%)	0.7	0.2	0.8
	1	17 (13%)	4 (17%)	13 (20%)			
**Dried legumes and nuts** n (%)	0	111 (87%)	18 (75%)	59 (94%)	0.2	0.2	**0.024***
	1	17 (13%)	6 (25%)	4 (6%)			
**Fruits** n (%)	0	98 (77%)	16 (67%)	51 (81%)	0.3	0.6	0.2
	1	30 (23%)	8 (33%)	12 (19%)			
**Whole grains** n (%)	0	119 (93%)	24 (100%)	49 (78%)	0.4	**0.004***	**0.009***
	1	9 (7%)	0 (0%)	14 (22%)			
**Lean meat** n (%)	0	88 (69%)	13 (54%)	45 (72%)	0.2	0.7	0.14
	1	40 (31%)	11 (46%)	18 (28%)			
**Fish** n (%)	0	122 (95%)	23 (96%)	60 (95%)	>0.9	>0.9	>0.9
	1	6 (5%)	1 (4%)	3 (5%)			
**Fatty meat and processed meat** n (%)	0	83 (65%)	11 (46%)	42 (67%)	0.11	0.9	0.089
	1	45 (35%)	13 (54%)	21 (33%)			
**High-fat, non-fermented dairy products** n (%)	0	69 (54%)	13 (54%)	29 (46%)	>0.9	0.4	0.6
	1	59 (46%)	11 (46%)	34 (54%)			
**Low-fat fermented dairy products** n (%)	0	107 (83%)	23 (96%)	54 (86%)	0.2	0.8	0.3
	1	21 (17%)	1 (4%)	9 (14%)			
**Vegetable oils** n (%)	0	119 (93%)	21 (88%)	60 (95%)	0.4	0.8	0.3
	1	9 (7%)	3 (12%)	3 (5%)			
**Olive and canola oil** n (%)	0	123 (96%)	24 (100%)	59 (94%)	>0.9	0.5	0.6
	1	5 (4%)	0 (0%)	4 (6%)			
**Avocado** n (%)	0	126 (99%)	24 (100%)	62 (99%)	>0.9	>0.9	>0.9
	1	2 (1%)	0 (0%)	1 (1%)			
**Sugar** n (%)	0	53 (41%)	17 (71%)	29 (46%)	**0.013***	0.6	0.054
	1	75 (59%)	7 (29%)	34 (54%)			

**Wine**	0	108 (84%)	22 (92%)	45 (71%)	0.5	0.053	**0.05***
n (%)	1	20 (16%)	2 (8%)	18 (29%)			

Legend: CD: celiac disease, MDS: Mediterranean Diet Score. Inadequate adherence (marked as 0) to MD was defined as an MDS of 0 or 0.5. Adequate adherence (marked as 1) to MD was defined as an MDS of 1. All percentages were rounded to the nearest whole number. Statistical test: Fisher's exact test, level of significance: $p<0.05$. Significant results are highlighted with bold numbers.
